# Metastatic Breast Cancer or Multiple Myeloma? Camouflage by Lytic Lesions

**DOI:** 10.1155/2010/509530

**Published:** 2010-03-07

**Authors:** Bruce Hough, Adam Brufsky, Suzanne Lentzsch

**Affiliations:** Department of Medicine, Division of Hematology/Oncology, University of Pittsburgh Cancer Institute, Pittsburgh, PA 15232, USA

## Abstract

We report a case of a female with stage I infiltrating ductal carcinoma who received adjuvant therapy including trastuzumab. One year later she developed lytic lesions and was retreated with trastuzumab that was held after she developed symptomatic heart failure. Lytic lesions were attributed to relapse of breast cancer, and cardiac failure attributed to prior trastuzumab therapy. After complications necessitated multiple hospitalizations, a further workup revealed that the lytic lesions were not metastatic breast cancer but multiple myeloma. Her advanced multiple myeloma was associated with systemic amyloidosis involving gut and heart, which ultimately led to her demise. This report addresses the pitfalls of overlapping symptoms and the question of which patients with suspected metastatic disease should undergo a biopsy.

## 1. Case Report

A 63-year-old white female was diagnosed with stage 1 infiltrating ductal carcinoma at an outside hospital in August 2005. Her tumor was 1 cm in size and was ER/PR/Her2Neu positive with an oncotype score of 29. Axillary lymph node sampling revealed 0/6 positive lymph nodes. She underwent adjuvant treatment with epirubicin, cyclophosphamide, and trastuzumab later that year. At that time she had no clinical signs of congestive heart failure (CHF). In January 2007, she developed pain in her sternum and back, and X-rays revealed multiple small lytic lesions. The diagnosis of metastatic breast cancer was made and she underwent radiation therapy to the symptomatic lesions as well as further trastuzumab treatment for metastatic disease. She did not receive hormones at the time of diagnosis of presumed relapse. Soon after start of adjuvant therapy, she developed symptomatic CHF and was found to have a left ventricular ejection fraction of 15%. The patient was diagnosed empirically with trastuzumab-induced cardiomyopathy at an outside hospital and was started on diuretics. After a time in rehab, she improved and went back home. 

In February 2008, she presented to our institution for a second opinion regarding metastatic breast cancer treatment. Because she was dehydrated and lightheaded she was admitted. The patient was diagnosed with renal failure with a creatinine above 3 mg/dL. Her work-up also included serum and urine protein electrophoresis. Both demonstrated a monoclonal gammopathy. She was noted to have 3.5 gm of protein on a 24-hour urine collection and both the urine and serum immunofixation demonstrated an IgG kappa monoclonal protein with a serum M-spike of.88 gm/dL. Her serum free light chains revealed a serum free kappa of 8300 mg/L with a kappa/lambda ratio of 418. Quantitative immunoglobulins were normal, Beta-2 microglobulin was 15.6 mg/L and calcium was normal. 

The inpatient hematology service was asked to comment on the seemingly unusual constellation of diagnoses: metastatic breast cancer, trastuzumab-induced cardiotoxicity, and a new diagnosis of multiple myeloma. She was noted to have bilateral pleural effusions, pedal edema, and nausea. Complaints of back pain led to the discovery of multiple thoracic compression fractures. Because of back pain and concern for cord instability, she underwent T5, T6, and T7 corpectomy with reconstruction. Pathologic evaluation of the resected bony fragments revealed no breast tumor cells, while some plasma cells were detected. There was not enough tissue to adequately evaluate for plasma cell clonality by immunohistochemistry. A bone marrow biopsy revealed clonal kappa light chain restricted cells comprising 30% of the total cells consistent with a diagnosis of multiple myeloma. Cytogenetics revealed monosomy 13. A bone survey showed progressing lytic lesions and a CT guided biopsy of a sacral lesion was performed that revealed numerous atypical plasma cells that stained positive for CD138 and negative for pankeratin consistent with a plasma cell neoplasm. She was therefore staged III in the International staging system (ISS), and stage IIIB in the Durie-Salmon staging system with extensive bone disease and was started on thalidomide, dexamethasone, and bortezomib with consideration for autologous hematopoietic stem cell transplant in the future depending on improvement of her performance status. After receiving her first dose of thalidomide, dexamethasone, and bortezomib, she required admission to the hospital for shortness of breath and was noted to have worsening bilateral pleural effusions. While admitted she also complained of persistent nausea. A gastric emptying scan revealed emptying of 138 minutes (normal less than 110). An echocardiogram revealed both left ventricular wall and septal wall thickness. Her septal thickness measured 1.5 cm (normal 0.7–1.2 cm). Additional findings included multiple areas of hypokineses, dilated inferior vena cava and right atrium felt by the echo-cardiographer to be consistent with amyloid heart disease. While hospitalized she had persistent pedal edema, pleural effusions, anasarca, and subsequent hypotension with diuretic use. She was treated for a time with milrinone without appreciable change. Eventually the decision was made to change the direction of her care to comfort. She was discharged to a hospice closer to her home.

## 2. Discussion

This case prompts an interesting question: which patients with suspected metastatic disease do you biopsy? Clayer and Duncan [1] attempted to answer that question by following 50 patients with localized carcinoma who presented with new bony lesions. In 9 patients (15%) the new bony lesions were different from the primary tumor. Two of those nine cases were found to have multiple myeloma, one patient breast cancer, and another melanoma as the primary tumor. 

Given that there are no clear guidelines about which patients and which tumors require biopsy for verification of metastases, each diagnosis of metastasis should be carefully evaluated for the likelihood of association with the primary tumor. Factors that may help decide whether the metastasis might have arisen from a secondary malignancy include the age of the patient, the underlying biology of the primary, the site, grade and radiographic characteristics of the metastatic lesion, the stage and grade of the primary, and tumor markers.

In our case advanced multiple myeloma of the patient was likely associated with amyloidosis of the gut which led to her persistent nausea and delayed gastric emptying, and amyloid deposition of the heart which ultimately led to her demise. The premature assumption of trastuzumab-induced cardiomyopathy impeded questioning the likelihood of other reasons for CHF. In retrospect, it may not have been the trastuzumab that contributed to her cardiomyopathy during treatment of her presumed breast cancer recurrence, but rather amyloidosis involving the heart and gut. In accordance with that are findings of the HERA study in which only 1.7% of the trastuzumab-treated patients developed symptomatic CHF [2]. This is similar to results of cardiotoxicity in 2% of women who received trastuzumab as a single agent for metastatic disease reported by Vogel et al. [3]. 

In order to provide an optimal treatment for metastatic disease, it is important to determine the likelihood of the origin of metastasis. While hypercalcemia and anemia are common in both diseases, the M-spike on the serum protein electrophoresis was the first indication that her lytic lesions were due to myeloma and not to breast cancer. In this case, a careful analysis of existing blood work might have provided valuable hints, such as elevated protein, for further diagnostic steps. Elevated protein in blood or urine should be further evaluated by serum and urine protein electrophoresis as well as free serum light chain ratio. Additionally, if there is extensive skeletal disease on standard X-rays with concomitant normal bone scans, multiple myeloma should be considered. While breast cancer metastases can have blastic and lytic lesions, myeloma bone lesions are purely osteolytic due to increased osteoclast activity and suppressed osteoblast activity [4]. Radiotracer is taken up only by activated osteoblasts and as such, bone scans are quite often negative even with extensive skeletal involvement by myeloma [5]. Bone-specific alkaline phosphatase in serum is an indicator of osteoblast activity [4]. Therefore, low levels of bone-specific alkaline phosphatase associated with high levels of bone resorption markers such as C-telopeptide (CTX) and N-telopeptide (NTX) are suggestive for myeloma bone lesions. 

In addition biopsies are important to provide optimal management of breast cancer patients with distant metastases. Patients with metastatic progression of local malignancies can acquire genetic defects that might alter the treatment plan. Specifically, in patients with breast cancer, the appropriate treatment varies according to both ER and Her-2-Neu receptor status. Simmons et al. noted a high discordance rate in 29 patients who underwent biopsy of metastatic lesions. The discordance rate between the primary and the metastatic site was 40% for hormonal status and 8% for Her-2-Neu status, changing the treatment plan in 20% of those patients [6, 7]. 

Taken together, this case emphasizes that it is essential to thoroughly analyze the likelihood of the origin of metastasis in each patient. To provide an optimal treatment for metastatic disease, biopsy should be considered unless absolutely contraindicated and a careful differential diagnosis should be generated and investigated if a biopsy is not possible.
